# NADPH Oxidases in Pain Processing

**DOI:** 10.3390/antiox11061162

**Published:** 2022-06-14

**Authors:** Wiebke Kallenborn-Gerhardt, Katrin Schröder, Achim Schmidtko

**Affiliations:** 1 Institute of Pharmacology and Clinical Pharmacy, Goethe University Frankfurt, Max-von-Laue-Str. 9, 60438 Frankfurt, Germany; schmidtko@em.uni-frankfurt.de; 2 Institute of Cardiovascular Physiology, Goethe University Frankfurt, 60590 Frankfurt, Germany; schroeder@vrc.uni-frankfurt.de; 3 German Center for Cardiovascular Research (DZHK), Partner Site Rhein Main, 60590 Frankfurt, Germany

**Keywords:** NADPH oxidase, Nox, pain, nociception, inflammation, peripheral injury, neuropathy, Nox inhibition

## Abstract

Inflammation or injury to the somatosensory nervous system may result in chronic pain conditions, which affect millions of people and often cause major health problems. Emerging lines of evidence indicate that reactive oxygen species (ROS), such as superoxide anion or hydrogen peroxide, are produced in the nociceptive system during chronic inflammatory and neuropathic pain and act as specific signaling molecules in pain processing. Among potential ROS sources in the somatosensory system are NADPH oxidases, a group of electron-transporting transmembrane enzymes whose sole function seems to be the generation of ROS. Interestingly, the expression and relevant function of the Nox family members Nox1, Nox2, and Nox4 in various cells of the nociceptive system have been demonstrated. Studies using knockout mice or specific knockdown of these isoforms indicate that Nox1, Nox2, and Nox4 specifically contribute to distinct signaling pathways in chronic inflammatory and/or neuropathic pain states. As selective Nox inhibitors are currently being developed and investigated in various physiological and pathophysiological settings, targeting Nox1, Nox2, and/or Nox4 could be a novel strategy for the treatment of chronic pain. Here, we summarize the distinct roles of Nox1, Nox2, and Nox4 in inflammatory and neuropathic processing and discuss the effectiveness of currently available Nox inhibitors in the treatment of chronic pain conditions.

## 1. Introduction

Inflammation, damage, or diseases affecting the somatosensory system may result in chronic pain associated with hypersensitivity to noxious stimuli (hyperalgesia), enhanced responses to normally innocuous stimuli (allodynia), or spontaneous pain. Currently available first-line pharmacotherapy options for chronic pain include nonsteroidal anti-inflammatory drugs (NSAIDs), opioids, or, for neuropathic pain, tricyclic antidepressants, selective serotonin-norepinephrine-reuptake inhibitors, or gabapentinoids [1,2,3,4]. However, in many patients with chronic pain, the treatment is only partially effective and/or associated with severe side effects. Thus, chronic pain is an enormous medical burden and there is a large unmet medical need to elucidate molecular mechanisms of pain processing in order to develop novel strategies for treatment of chronic pain [5,6,7]. 

Hypersensitivity to mechanical and thermal stimuli are mediated by various alterations in the peripheral as well as in the central nervous system [5,8,9]. Emerging lines of evidence suggest that reactive oxygen species (ROS) are generated in somatosensory pathways and essentially contribute to the development of pain hypersensitivity in a specific manner. For example, systemic or intrathecal treatment with ROS scavengers or antioxidants attenuated the hypersensitivity in different animal models of chronic inflammatory and neuropathic pain as well as in cancer-induced bone pain [10,11,12,13,14,15,16,17,18,19,20,21,22,23,24,25,26]. In line with this, several studies have demonstrated exaggerated pain behavior in response to treatment with ROS donors such as tert-butyl-hydroperoxide (TBHP) [16,19,21,23,27] or inactivation of antioxidant enzymes such as superoxide dismutase (SOD) 1 and 2 or sestrin 2 [23,28,29]. ROS are highly reactive and thus can potentially damage nucleic acids, proteins, and lipids, especially at high concentrations [30]. However, recent studies have demonstrated that ROS at physiological concentrations mediate reversible regulatory processes, for example, by targeting redox-sensitive cysteine residues of proteins. Importantly, in this context, ROS may serve as functional messenger molecules, whose in vivo relevance is being increasingly recognized [31]. Indeed, specific functions of reversible ROS signaling have been demonstrated in various physiological settings including the nociceptive system [32,33,34,35]. Redox-dependent activation of ion channels such as transient receptor potential melastatin subtype 2 (TRPM2) or transient receptor potential A1 (TRPA1), redox-dependent activation of kinases such as cGMP-dependent protein kinase Iα (PKG Iα, synonym: cGKIα), or redox-dependent activation of transcription factors such as NF-E2-related factor 2 (Nrf2) have been identified as specific ROS targets in nociceptive signaling [36,37,38,39]. For example, specific signaling of ROS is achieved by targeting distinct cysteine residues of TRPA1, modulating the activation of the channel (for review, see [38]), or inducing a redox-dependent dimerization of PKG Iα, leading to an increased activity of this kinase, subsequently affecting neuropathic pain processing [36,40,41]. 

Important ROS sources in the nociceptive system include mitochondria, xanthine oxidase, and nicotinamide adenine dinucleotide phosphate (NADPH) oxidases [42,43,44,45,46,47,48]. In this review, the focus is set on the function of NAPDH oxidases of the Nox family in pain processing. Nox family members are membrane-bound enzyme complexes, which consist of different subunits and whose main function is the production of ROS [46,49,50,51,52]. This is in contrast to other ROS-generating enzymes, such as xanthine oxidase, cyclooxygenase, or lipoxygenases, which produce ROS as a byproduct of their normal catalytic activity. The Nox family of NADPH oxidases consists of seven members: Nox1–5 and the dual oxidases Duox1 and 2. Importantly, subunit composition as well as expression patterns differ between different NADPH oxidases, and in line with this, they have been described to regulate various physiological and pathophysiological processes such as immune defense, cardiovascular processes, neurodegeneration, and pain processing [46,49,53,54,55,56]. As Nox5 is absent in mice and rats, there are only limited data available concerning the physiological function of Nox5 [57]. As mentioned above, the only proven metabolic function of NADPH oxidases is the production of ROS via cross-membrane electron transfer from NADPH to oxygen as an electron acceptor, resulting in the production of superoxide or hydrogen peroxide (for review, see [50,51,58]). Thus, NADPH oxidases seem to alter physiological and pathophysiological pathways by local production of ROS that probably modulate enzyme and ion channel activity. The functional roles of NADPH oxidases in various diseases have been summarized in excellent recent reviews focusing on the cardiovascular system, infectious and inflammatory diseases, neurodegenerative disorders, and the peripheral nervous system [46,55,56,59,60]. In the nociceptive system, distinct functional roles of ROS produced by Nox1, Nox2, and Nox4 have been identified [43,46,58]. Therefore, we will herein describe the emerging functions of NADPH oxidases in pain processing and highlight the distinct contributions of Nox1, Nox2, and Nox4 to different types of chronic pain.

## 2. NADPH Oxidases Affect Pain Processing by Distinct Mechanisms

### 2.1. Nox2

**Figure 1:**
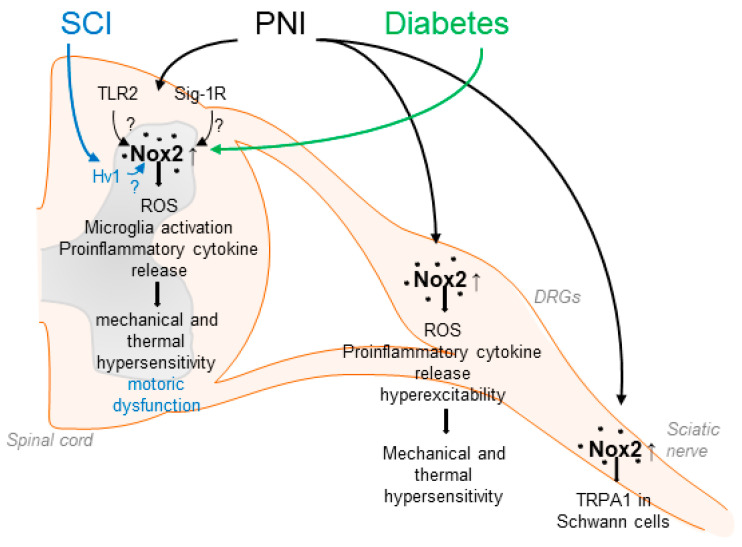
Suggested contributions of Nox2 to pain processing in response to peripheral nerve injury (PNI), spinal cord injury (SCI) and diabetic neuropathy. Nox2 activity is increased in the spinal cord, DRGs and the sciatic nerve after up-regulation of Nox2 expression in microglial cells or through recruitment of Nox2-positive macrophages. This leads for example to enhanced ROS production and enhanced release of proinflammatory cytokines, resulting in enhanced mechanical and thermal hypersensitivity. Activation of Nox2 in response to PNI has been suggested to be mediated by TLR2 and Sig-1R. In addition, SCI and diabetic conditions also lead to enhanced Nox2 expression and activity in the spinal cord.

Nox2 was the first NADPH oxidase described, originally cloned as the catalytic subunit of the phagocyte NADPH oxidase [61,62]. Nox2 is expressed in various types of cells and tissues including the peripheral and central nervous system [33,46,58]. The activity of Nox2 is tightly controlled by the translocation of the regulatory subunits p40phox, p47phox, p67phox, and the small guanosine triphosphate (GTP)ase Rac to the membrane-bound subunits p22phox and Nox2, leading to a conformational change, which allows electron transport from NADPH over the membrane to reduce molecular oxygen [49]. Several studies have demonstrated that Nox2 is involved in neuropathic pain processing. For example, Nox2 expression is induced in the spinal cord and DRGs in different rodent models of peripheral nerve injury [29,63,64,65] and spinal cord injury [66]. Nox2 expression in the spinal cord seems to be restricted to microglia and is absent in neurons [63], while in dorsal root ganglia (DRGs), Nox2 has been detected in macrophages and neurons after injury [64,65,67]. Notably, spinal ROS production, microglia activation, and production of proinflammatory cytokines, such as TNF-α or IL-1β, were ameliorated in Nox2-deficient mice after peripheral nerve injury. Moreover, mechanical and thermal hypersensitivity after peripheral nerve injury were attenuated in the absence of Nox2, suggesting an essential functional role of Nox2-mediated ROS production during neuropathic pain [63,64] (Table 1). Another study observed that Nox2 activation in spinal microglia depends on Toll-like receptor 2 (TLR2) because nerve injury-induced upregulation of Nox2, spinal microglia activation, and ROS production were abrogated in TLR2-deficient mice [68]. Conversely, the activation of TLR2 resulted in increased Nox2 expression, increased microglia activation, and ROS production in the spinal cord [68]. Supporting the findings described above, in a recent study, high-frequency stimulation (HFS) of the sciatic nerve resulted in Nox2 upregulation in the lumbar spinal cord, whereas Nox2 inhibition via a blocking peptide or shRNA delivery attenuated HFS-induced mechanical allodynia and activity of dorsal horn neurons [69] (Figure 1). 

In addition to affecting signaling pathways in the spinal cord, Nox2 is involved in neuropathic pain processing in the peripheral nervous system [64]. Several studies revealed that, in response to peripheral nerve injury, Nox2-expressing macrophages are recruited to DRGs, thereby promoting ROS production and upregulation of TNF-α [64,65]. The importance of macrophages for pain processing is supported by several studies, in which an essential role of DRG macrophages in the development and attenuation of neuropathic pain has been uncovered [77,78,79,80]. As TNF-α receptors are expressed in DRG neurons, these data imply that macrophage–neuron signaling in response to peripheral nerve injury at least partly depends on Nox2 [64]. The functional relevance is also supported by a recent study in which inhibition of Nox2 in DRGs using gp91-tat or Nox2-shRNA after peripheral nerve injury attenuated hyperexcitability of DRG neurons and plasma membrane translocation of PKCε [65]. Moreover, Logu et al. showed that Nox2-expressing macrophages, which are recruited to the injured site after nerve injury, activate TRPA1 in Schwann cells, which in turn results in Nox1 activation and contributes to mechanical allodynia [81] (Figure 1). The accumulation of macrophages in DRGs was also recently observed in a model of osteoarthritis pain [82]. Hence, the recruitment of Nox2-positive macrophages and increased ROS production in DRGs seems to be a general mechanism of persistent pain processing. 

The question arises which mechanisms lead to an enhanced Nox2 activity after peripheral nerve injury. A possible upstream mechanism, in addition to the aforementioned regulation by TLR2, involves the activation of sigma-1 receptors (Sig-1Rs), a group of transmembrane receptors predominantly localized to the endoplasmic reticulum. Activation of Sig-1R by its agonist PRE084 resulted in increased spinal Nox2 activity (demonstrated by translocation of p47phox to the membrane), increased ROS production, and enhanced mechanical allodynia and thermal hyperalgesia. Similar results were obtained after peripheral nerve injury, suggesting that Sig-1R might drive Nox2 activation in this setting [83]. A follow-up study of this group further indicated that neuronal nitric oxide synthase (nNOS) might function as a link between spinal Sig-1R and Nox2 [84] (Figure 1). 

Accumulating evidence indicates that Nox2 is also involved in pain signaling in response to spinal cord injury, a devastating event that often results in motor dysfunction below the level of lesion and chronic debilitating pain. Sabirzhanov et al. reported that Nox2 expression is increased in spinal microglia after spinal cord injury and that inhibition of Nox2 using NOX2ds-tat or deletion of Nox2 in knockout mice reduced the ROS production, motor dysfunction, and hypersensitivity [72]. Furthermore, recent studies have suggested that the phagocyte-specific proton channel Hv1 might act upstream of Nox2 in this model, because Hv1-deficiency was associated with decreased Nox2 expression and ROS production, reduced tissue acidosis, and improved locomotor function after spinal cord injury [85,86]. Interestingly, an earlier study demonstrated that Hv1 deficiency in neutrophils leads to disturbed proton currents, calcium signals, and neutrophil motility. These findings suggest that Hv1 channels compensate the charge induced by Nox2 and maintain calcium entry signals required for intact neutrophil function [87]. 

A functional role of Nox2 has been detected in diabetic neuropathy, a serious and common complication of type 1 and type 2 diabetes. Nox2 and p47phox are upregulated in the spinal cord in a streptozotocin-induced type 1 diabetic model in rats associated with neuropathic pain. In line with this, mechanical hypersensitivity was decreased by treatment with the unspecific Nox2 inhibitor apocynin [88]. Further work also suggested a contribution of Nox2/ROS signaling to the development of neuropathic pain in a model of type 2 diabetes, as Nox2 expression was induced in the spinal cord of type 2 diabetic rats and treatment with the ROS scavenger α-phenyl-N-tert-butyl nitrone attenuated diabetic neuropathic pain behavior in response to mechanical and thermal stimuli [26]. These results are in agreement with earlier studies that showed that ROS contribute to hyperglycemia-induced neuronal damage [46,89,90,91]. Moreover, a pronociceptive role of Nox2 has been suggested in cancer-induced bone pain, which is a mixed-mechanism pain state (both neuropathic and inflammatory) caused by bone metastases or primary bone tumors. Nox2 expression and ROS levels were elevated in the spinal cord of rats in a model of cancer-induced bone pain, and treatment with apocynin attenuated the pain hypersensitivity [92]. However, only apocynin, a non-selective and rather unspecific Nox inhibitor, whose Nox2-inhibiting activity depends on the presence of myeloperoxidase [93], was used. As myeloperoxidase is only expressed in a small subpopulation of spinal cord neurons and is absent in spinal microglia [67], further studies using Nox2-deficient mice or targeting Nox2 specifically need to be carried out. 

In addition to the aforementioned pronociceptive functions, Nox2 seems to be involved in opioid-induced antinociceptive tolerance. Opioid tolerance is characterized by a reduced responsiveness to an opioid agonist, such as morphine, and usually requires increasing doses to achieve the desired effect [94]. In a model of morphine-induced tolerance, Nox2 expression was upregulated in the spinal cord after chronic morphine administration [95]. Mice lacking Nox2 or its catalytic subunit p47phox developed antinociceptive tolerance in the first 3 days after continuous morphine treatment; however, at later time points, morphine analgesia was restored in Nox2-deficient mice. In line with this, increased spinal Nox activity, anti-inflammatory cytokine production, and nitration of MnSOD, which was induced in this model, were attenuated in Nox2-deficient mice [73], suggesting that Nox2 activity promotes opioid-induced antinociceptive tolerance. 

Overall, many of the above-described studies point to an important function of Nox2 in neuropathic pain processing. However, whether inhibition of Nox2 might have a therapeutic potential in the treatment of neuropathic pain or other pain conditions needs to be elucidated in further studies. Nonetheless, one also has to consider the fact that Nox2-dependent superoxide production is essential for an appropriate host defense and that it plays a critical role in the resolution of inflammation [49,60,96,97,98,99]. Hence, Nox2-inhibiting therapeutic strategies might be associated with various unwanted side effects.

### 2.2. Nox1

**Figure 2:**
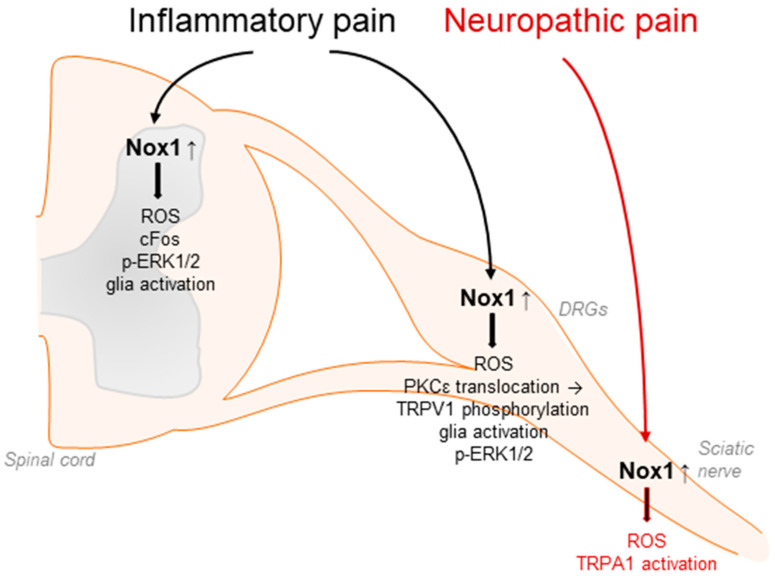
Nox1 affects inflammatory pain processing by redox regulation and translocation of PKCε to the plasma membrane. This subsequently modulates TRPV1-mediated signaling, glia activation, cFos upregulation, and phosphorylation of ERK1/2 in DRGs and/or in the spinal cord. In addition, Nox1 expressed in Schwann cells affects neuropathic pain signaling in response to peripheral nerve injury through activation of TRPA1 in sensory neurons.

Accumulating evidence suggests that Nox1 is involved in the processing of inflammatory and neuropathic pain as well as migraine-related pain. In general, Nox1 activity is controlled by the binding of p22phox, Rac, and the cytosolic subunits Nox organizer 1 and Nox activator 1, homologues of p47phox and p67phox, respectively. Nox1 is predominantly expressed in the colon, prostate, uterus, and vascular cells and has been linked to cardiovascular diseases such as atherosclerosis or hypertension [49,51,56,100,101,102,103]. Moreover, Nox1 has been detected in DRG lysates, in spinal cord glia and neurons, and in Schwann cells [33,67,71,81]. Nox1-deficient mice exhibited significantly attenuated pain behavior in the second phase of the formalin test, the acetic acid-induced writhing test, and the carrageenan-induced mechanical and thermal hyperalgesia [70] (Table 1). Similar results were obtained using the Nox1 inhibitor ML171 [104]. The administration of ML171 ameliorated the nociceptive behavior in both phases of the formalin test and reduced the formalin-induced ROS production, c-Fos upregulation, ERK1/2 phosphorylation, and glia activation in DRGs and the spinal cord [104,105]. Further, Nox1 promotes the translocation of protein kinase C (PKC) ε and subsequent phosphorylation of transient receptor potential (TRP)V1 channel during inflammatory pain processing [70] (Figure 2).

Nox1 is also expressed in Schwann cells of the sciatic nerve. Inhibition of Nox1 activity by GKT137831 or ML171 effectively blocked TRPA1-mediated ROS production in cultured Schwann cells as well as peripheral nerve-injury-induced allodynia and neuroinflammation [81]. Interestingly, in this context, Nox1 activity seems to be stimulated by ROS from Nox2-expressing macrophages, which activate TRPA1 and Nox1 in Schwann cells, finally leading to sensitization processes in peripheral neurons. These results were confirmed using antisense oligonucleotides directed against Nox1 [81] (Figure 2).

In addition, a role of Nox1 in glyceryl-nitrate-induced migraine-related pain and ethanol-evoked neuropathic pain processing has been described [81,106]. Moreover, Nox1 might play a function in morphine-induced analgesia and tolerance, because in Nox1-deficient mice, the analgesic effects of morphine were enhanced and analgesic tolerance was suppressed compared to WT mice [71].

### 2.3. Nox4

**Figure 3:**
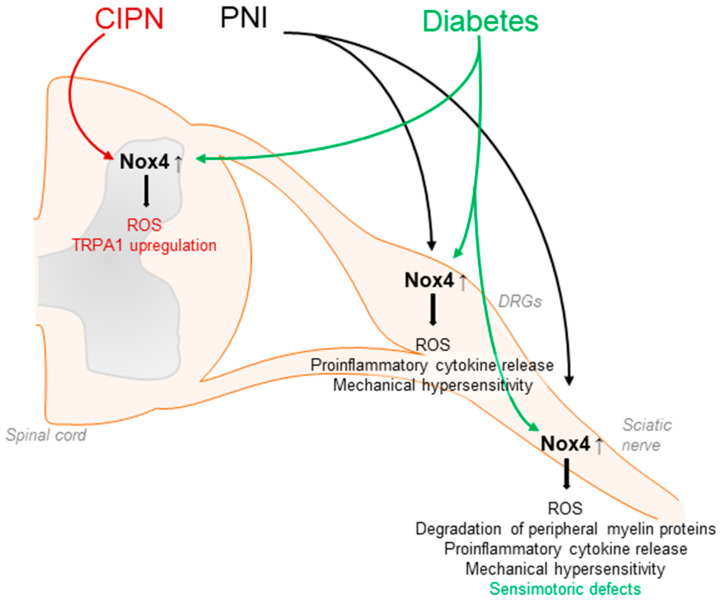
Contribution of Nox4 to neuropathic pain signaling. Nox4 is expressed in primary afferent neurons and Nox4-derived ROS induce the degradation of peripheral myelin proteins in peripheral nerves after injury, leading to mechanical hypersensitivity. Similar effects are observed in diabetic models. Furthermore, Nox4 promotes ROS production and proinflammatory cytokine release in DRGs and the sciatic nerve. CIPN: chemotherapy-induced peripheral neuropathy; PNI: peripheral nerve injury.

Nox4 seems to have a major function in neuropathic pain processing. Nox4 has been detected in neuronal subpopulations of the nociceptive system, including sensory neurons and inhibitory interneurons of the spinal cord [67,74]. In general, Nox4 activity requires only p22phox as a cofactor and, unlike the other Nox isoforms, Nox4 is constitutively active, although its activity might be further regulated by other factors such as protein disulfide isomerase (PDI) or Poldip2 [51]. Nox4 is highly expressed in proximal tubular cells of the kidney, endothelial cells, fibroblasts, and osteoclasts, and may exert damaging or protective effects in a cell-specific manner. Furthermore, Nox4 signaling has been linked to pulmonary diseases as well as to various types of cancer [49,51,56,107,108,109,110,111,112]. Interestingly, in response to peripheral nerve injury or diabetic neuropathy, Nox4 expression is increased in the injured nerve, DRGs, and/or the spinal cord [75,113]. Notably, neuropathic pain behavior after peripheral nerve injury was attenuated in global Nox4 knockout mice [74,75], after temporal somatic knockdown of Nox4 in tamoxifen-inducible Nox4 knockout mice and in tissue-specific knockout mice lacking Nox4 in sensory neurons [27,74] (Table 1). Nox4 is supposed to affect pain by various mechanisms. Nerve-injury-induced ROS production was considerably reduced in the peripheral nerves of Nox4-deficient mice. In parallel, injury-induced degradation of peripheral myelin proteins such as myelin protein zero (MPZ) or peripheral myelin protein 22 (PMP22) as well as structural changes in the injured nerve were less pronounced in Nox4-deficient mice [74]. As changes in peripheral myelin may result in the sensitization of peripheral nerves and spontaneous action potentials, these alterations promoted by Nox4 might contribute to the manifestation of neuropathic pain [114,115,116,117]. In line with this, action potential firing in DRG neurons after peripheral nerve injury was reduced in the absence of Nox4 [27], and the inhibition of Nox4 by GKT137831 in a model of hyperglycemia-induced neurophysiological disorders restored expression of peripheral myelin proteins and prevented sensorimotor defects in mice, further suggesting a contribution of Nox4 in diabetes-induced peripheral neuropathy [118]. Another study revealed that the induction of proinflammatory cytokines such as TNFα and ROS production induced by peripheral nerve injury was less pronounced in Nox4 knockout mice [75]. Additional work has indicated that Nox4 might trigger the activation of JNK and DRG neuron apoptosis by activation of caspase 3 and PARP-1 [119]. Furthermore, in peripheral nerves, Nox4 regulates the expression of the Ca^2+^ binding protein S100A4, which seems to play an inhibitory function in neuropathic pain processing [27] (Figure 3). 

Nox4 has also been implicated in chemotherapy-induced peripheral neuropathy, because the downregulation of Nox4 suppressed the upregulation of TRPA1 in the spinal cord in a model of oxaliplatin-induced peripheral neuropathy [120]. Furthermore, in a model of cancer-induced bone pain, Nox4 expression was induced in spinal microglia, and the downregulation of Nox4 by intrathecal lentiviral injection attenuated bone-cancer-induced pain behavior. In accordance with the behavioral effects, the upregulation of nNOS and NMDAR2D was reversed by the Nox4 knockdown, while SOD and GABAA-γ2 downregulation was partly rescued [121]. 

A recent finding is that Nox4 also affects pain processing by the regulation of descending inhibitory pain pathways [122]. In a model of Parkinson’s disease (PD), Nox4 expression was significantly upregulated in the midbrain periaqueductal gray, and blocking Nox4 in this area by infusion of the Nox4 inhibitor GKT137831 resulted in decreased oxidative stress and blunted mechanical and thermal pain responses. Moreover, GKT137831 treatment also restored GABA-concentrations in the periaqueductal gray in a model of PD [122]. 

Unlike its relevance in neuropathic pain processing, Nox4 does not seem to be involved in the processing of acute nociceptive and inflammatory pain. Nox4-deficient mice showed a similar behavior as compared to their wildtype littermates in various acute nociceptive and inflammatory pain models, such as the hot plate test, formalin test, or inflammatory hyperalgesia induced by intraplantar injection of zymosan or CFA [74].

## 3. Nox Isoforms as Potential Analgesic Targets

As mentioned above, various ROS scavengers and antioxidants provided analgesic effects in animal models of persistent pain. However, as ROS also act as cell signaling molecules in physiological signaling pathways and metabolic processes [123], a general antioxidant therapy might be associated with unwanted side effects. Hence, specifically targeting ROS sources such as Nox isoforms might serve as a novel and more promising therapeutic approach in treating chronic pain conditions. 

Many studies investigating the functional role of NADPH oxidases have been carried out using non-specific Nox inhibitors such as diphenyleneiodonium (DPI) or apocynin, compounds that inhibit flavin-adenine-dinucleotide-containing enzymes such as nitric oxide synthase, or acting as antioxidants [109,124]. In the last decade, the development of specific Nox inhibitors has come into the focus of research and various excellent reviews are available characterizing the chemical structure and properties of these compounds [109,124,125,126]. 

### 3.1. The Triazolo Pyrimidines VAS2890 and VAS3947

**Figure 4:**
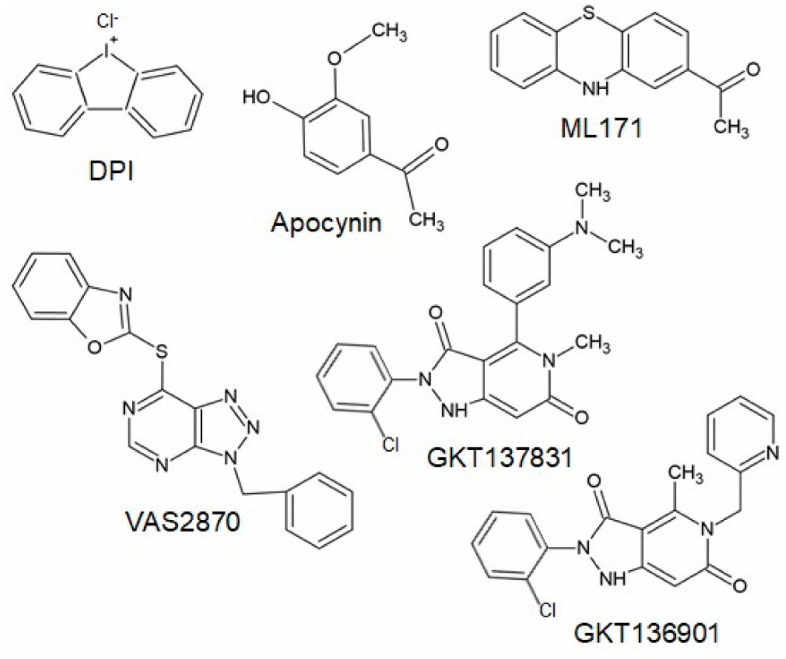
Chemical structures of potential Nox inhibiting compounds (abstracted from [125,133]).

The triazolo pyrimidines VAS2870 and VAS3947 (Figure 4) are pan-Nox inhibitors. VAS2870 displays rapid and reversible Nox inhibiting activity without inhibiting xanthine oxidase or showing ROS scavenging activities [127]. IC_50_ values have been published only for Nox2 (0.77 µM); however, other studies showed that Nox4 and Nox5 activity is also inhibited by this compound [102,125,128]. VAS3947, a VAS2870 analog, displays higher solubility and inhibits Nox1, Nox2, and Nox4 with similar IC_50_ values in cell-free assays without inhibiting xanthine oxidase or eNOS [125,129]. Thus, VAS2870 and VAS3947 can be considered to be Nox-specific; however, they apparently do not act in a Nox isoform-selective manner [109,125,127,128,129,130]. So far, neither VAS2870 nor VAS3947 have been tested in animal models of persistent pain conditions; however, several in vitro studies using VAS2870 are available, supporting the above-described in vivo findings of the involvement of NADPH oxidases in pain processing. For example, VAS2870 inhibited ROS production induced by TNFα and cyclin-dependent kinase 5 in HEK293 cells and primary cultures of sensory neurons [131]. Further in vivo studies showed that VAS2870 ameliorates the blood–brain barrier damage and neuronal damage in models of ischemic stroke or hemorrhagic transformation and Huntington’s disease [128,132]. As Nox1, Nox2, and Nox4 have been shown to be inhibited by VAS2870 and VAS3947, it seems likely that these compounds might also attenuate chronic pain conditions in vivo. However, it should be considered that VAS2780 can evoke calcium responses in TRPA1-transfected HEK cells, suggesting an activation of TRPA1 independent from NADPH oxidases that might limit the analgesic efficacy [133].

### 3.2. ML171

ML171 (Figure 4) is a compound described to inhibit Nox1 in a nano-molar range without inhibiting other ROS-producing enzymes or other Nox isoforms [134,135,136]. However, other studies demonstrated that ML171 rather interferes with the assay used to study NADPH oxidase activity; thus, results obtained using ML171 should be interpreted carefully [137,138]. As described above, a recent study demonstrated that the administration of ML171 attenuated formalin-induced nociceptive behavior and activity markers in tissue sections [104,105]. ML171 also attenuated mechanical hypersensitivity and macrophage recruitment to the injury site after peripheral nerve injury [81]. 

### 3.3. gp91ds-tat

A well-characterized peptidic inhibitor of Nox2 is gp91ds-tat, which shows high selectivity for Nox2 compared to Nox1 and Nox4 in a cell-free assay [125,139]. Gp91ds-tat has been used in several in vivo settings, including a model of spinal cord injury-induced neuropathic pain [72]. As described above, treatment with gp91ds-tat attenuated spinal cord injury-induced locomotor dysfunction and pain hypersensitivity [72]. Furthermore, the efficacy of gp91ds-tat treatment in neuropathic pain conditions was confirmed in another study [65].

### 3.4. Pyrazolopyridine Derivates GKT136901 and GKT137831

Another group of Nox inhibitors are pyrazolopyridine derivates. Among them, the Nox1/Nox4 inhibitor GKT137831 (Figure 4) is the most advanced compound and has already reached phase II of clinical trials for the treatment of type 1 diabetes mellitus [140]. Interestingly, GKT137831 attenuated paclitaxel-induced neuropathic pain behavior in rats [69] as well as pain sensitivity in a model of Parkinson’s disease [122], suggesting that this compound might be also effective in other pain conditions. However, GKT136901 (Figure 4), another pyrazolopyrimidine derivate that is supposed to inhibit Nox1 and Nox4, was ineffective in attenuating pain behavior induced by peripheral nerve injury in mice [75]. In contrast to studies described above, additional work characterizing Nox inhibitors revealed that GKT136901 rather interferes with the assay used to study NADPH oxidase activity and does not meet pharmacological criteria for a bona fide Nox inhibitor [136,138]. Recently, various other Nox-specific inhibitors such as GLX351322, GLX481372, GLX7013114, GSK2795039, CPP11G, or CPP11H have been identified as acting selectively on different Nox isoforms (for review, see [109,124]). Further studies need to be carried out in order to determine their efficacy in different pathophysiological settings in vivo.

## 4. Conclusions

Several lines of evidence demonstrate that ROS derived from NADPH oxidases are involved in the processing of various chronic pain conditions. Among them, the Nox isoforms Nox1, Nox2, and Nox4 are expressed in different cells of the nociceptive system and contribute to inflammatory and/or neuropathic pain processing via distinct signaling pathways. As oxidative stress caused by NADPH oxidases has been linked to various pathophysiological processes including chronic pain, and general antioxidative therapies show only insufficient evidence in clinical trials [109], targeting Nox isoforms might be a novel strategy for the treatment of chronic pain conditions. Even though the efficacy of the currently available selective Nox inhibitors for the treatment of pain needs to be elucidated in further studies and carefully evaluated concerning drug specificity, potential side effects, administration route, and treatment duration [109], inhibition of distinct Nox isoforms might complement currently available treatments, hopefully reducing side effects and leading to an effective pain relief.

## Figures and Tables

**Table 1:** Nox isoforms involved in pain processing.

Nox Isoform	Pain Type	Pain Model	Modality	Knockout Phenotype	Reference
Nox1	Nociceptive pain	-	thermal, heat	-	[70]
		-	mechanical	-	
		Formalin (phase 1)	chemical	-	
	Inflammatory pain	Formalin (phase 2)	chemical	↓	[70]
		Acetic acid	chemical	↓	
		Carrageenan	mechanical	↓	
		Carrageenan	thermal, heat	↓	
	Morphine analgesia	-	thermal, heat	↑	[71]
		-	mechanical	↑	
	Morphine- induced analgesic tolerance	-	thermal, heat	↓	[71]
Nox2	Neuropathic pain	Spinal nerve transection	thermal, heat	↓	[63]
		Spinal nerve transection	mechanical	↓	[63]
		Spared nerve injury	mechanical	↓	[64]
		Spinal cord injury	mechanical	↓	[72]
		Spinal cord injury	thermal, heat	↓	[72]
	Morphine- induced analgesic tolerance	-	thermal, heat	↓	[73]
Nox3	Not expressed in DRG neurons or spinal cord neurons; no data available on its role in pain processing				[67,70,74]
Nox4	Nociceptive pain	-	thermal, heat	-	[74]
		-	thermal, cold	-	
		Formalin (phase 1)	chemical	-	
	Inflammatory pain	Formalin (phase 2)	chemical	-	[74]
		Zymosan	mechanical	-	
		Complete Freund’s adjuvant	mechanical	-	
	Neuropathic pain	spared nerve injury	mechanical	↓	[27,74]
		Chronic constriction injury	mechanical	↓	[74,75]
		Chronic constriction injury	thermal, heat	-	[75]
Nox5	Absent in rodents				[76]
Duox1	Not expressed in the nociceptive system				[67]
Duox2	Expressed in DRG neurons, but no data available on its role on pain processing				[67]

Knockout phenotype as compared to WT mice: - not altered, ↓ reduced and ↑ increased.
